# Methotrexate as combination partner of TNF inhibitors and tocilizumab. What is reasonable from an immunological viewpoint?

**DOI:** 10.1007/s10067-015-2861-x

**Published:** 2015-01-22

**Authors:** Torsten Witte

**Affiliations:** Clinic for Immunology and Rheumatology, Medical University of Hannover, Carl-Neuberg-Str. 1, 30625 Hannover, Germany

**Keywords:** Cytokines and inflammatory mediators, Immunology, Rheumatic diseases, Rheumatoid arthritis (RA), Specialty fields, Tissues or models

## Abstract

The goal of therapy of rheumatoid arthritis is to achieve a remission or at least low disease activity. TNF inhibitors induce high remission rates only in combination with methotrexate, whereas the efficacy of tocilizumab is optimal even as a monotherapy. In this article, the differing dependence of the biological drugs on methotrexate is explained from the viewpoint of an immunologist. A selective search and evaluation of the literature was performed with regard to the mode of action of TNF inhibitors, tocilizumab and methotrexate in rheumatoid arthritis. Methotrexate primarily inhibits the activation and proliferation of lymphocytes. TNF inhibitors suppress monocytes and myeloid dendritic cells, and tocilizumab has a broader activity and is directed against both the lymphoid as well as the myeloid compartment. In view of the broad mode of action of tocilizumab, it can be explained why this drug, in contrast to TNF inhibitors, is acting optimally even in monotherapy.

## Background

Since the introduction of biologics into the treatment of rheumatoid arthritis (RA), patients’ symptoms have been significantly reduced and the process of joint destruction markedly decelerated. At present, about 15 % of RA patients in Germany are treated with biologics. Five TNF inhibitors, together with tocilizumab and abatacept, are approved for use as first-line biologics following failure of at least one or two conventional disease-modifying anti-rheumatic drugs (DMARDs). Earlier studies, e.g. the TEMPO trial for etanercept [1] and the PREMIER trial for adalimumab [2], showed that the TNF inhibitors in monotherapy are much less effective than in combination with methotrexate. In contrast, two studies (Charisma [3] and ACT-RAY [4]) demonstrated for tocilizumab monotherapy near equivalence to similar efficacy compared to tocilizumab in combination with methotrexate. In the *Ada*limumab *Act*emr*a* (ADACTA) trial, adalimumab and tocilizumab were compared in RA patients intolerant of methotrexate [5]. Tocilizumab was significantly superior to adalimumab as assessed by various outcome parameters, including ACR20 response and reduction of DAS28, CDAI and SDAI. In contrast to tocilizumab, TNF inhibitors require combination therapy with methotrexate for full effect. This article provides an overview of studies on the mode of action of TNF inhibitors, tocilizumab and methotrexate, and offers an explanation for the divergent dependency of TNF inhibitors and tocilizumab on the combination with methotrexate.

### Pathophysiology of rheumatoid arthritis

The initiation of RA is facilitated by a genetic predisposition. In addition, the probability of developing RA is influenced by environmental factors such as smoking, alcohol and nutrition. The autoimmune aspect of the disease begins many years before overt arthritis occurs. In this ‘pre-arthritis’ phase, the autoantibodies rheumatoid factor (RF) and anti-citrullinated peptide/protein antibodies (ACPA) can usually be detected [6]; however, even on biopsy, no inflammatory changes can be found in the joint [7]. At present, it remains unclear where the location of this pre-arthritis stage is. Candidates include the respiratory system (since RA affects mainly smokers) and the lymph nodes.

The joint inflammation, which usually starts after a long period of pre-arthritis, has three main phases:

### Adhesion and migration

At the initiation of arthritis, cells of the immune system move into the joints. Arthritis is probably triggered by an autoantigen in the joint. This autoantigen has not yet been identified, and triggers are likely to vary from patient to patient. Initially, antigen-presenting cells in the joint loaded with an autoantigen probably move to the central lymphatic organs and activate T cells there. They then migrate back to the joints, along with circulating immune cells.

### Activation/inflammation

Most of the lymphocytes in the synovial membrane comprise CD4^+^ T-helper cells that are mainly part of the Th1 and proinflammatory Th17 subsets [8, 9]. B cells and macrophages are also present, as well as large numbers of neutrophil granulocytes in the synovial fluid.

The cells of the immune system interact in the inflamed joint and activate each other by cell-cell contact as well as by the production of cytokines. The formation of cytokines such as TNF-α and IL-6 at the site of inflammation leads to activation of the endothelium cells in newly formed vessels and to an increase in adhesion receptors such as intercellular adhesion molecule 1 (ICAM-1) and vascular cell adhesion molecule 1 (VCAM-1) [10]. This further increases the adhesion and finally migration of leucocytes and lymphocytes from the blood into the inflamed joints [11].

### Destruction of the joint

Fibroblast-like synovial cells are activated and produce collagenases (such as MMP-1, MMP-3 and MMP-13), which attack the cartilage [12]. Then, an inflammatory pannus forms, in which T and B lymphocytes, macrophages and dendritic cells can be identified. The inflammatory tissue invades deeper into the cartilage and finally the bone. Cytokines formed in the ongoing inflammation, such as IL-6 and TNF-α, activate chondro- and osteoclasts and thus further contribute to the disintegration of bone and cartilage. Activated T cells and IL-18-stimulated macrophages produce receptor activator of nuclear factor kappa-B ligand (RANK-L), a substance that also activates osteoclasts and promotes the disintegration of bone. The inflamed tissue activates angioneogenesis with factors such as vascular endothelial growth factor (VEGF), prostaglandins, IL-8, ENA-78 or angiopoietin-1 [13, 14] in order to be sufficiently supplied with blood. In spite of the active formation of new vessels, blood supply to the inflammatory tissue is critical, and the pO_2_ in the synovial fluid is always markedly below that in the blood. Angioneogenesis therefore is essential for the perpetuation of the inflammation [15].

Insights into the pathophysiology of RA have led to the development and use of biologics. The mode of action of classical DMARDs, and specifically that of methotrexate, can now be at least partially explained.

## Mode of action of methotrexate

Methotrexate (MTX) was first used in the treatment of RA in 1951 [16], but has only been widely prescribed since the 1980s. Methotrexate is a folic acid antagonist which reduces the concentration of intracellular folinic acid needed for purine and pyrimidine metabolism as well as for amino acid synthesis. Methotrexate therefore has an anti-proliferative effect. Based on recent studies, methotrexate also increases the extracellular concentration of the anti-inflammatory molecule adenosine by dephosphorylation of adenine nucleotides, and therefore has an anti-inflammatory effect in addition to its anti-proliferative action.

### Activation/inflammation

**Fig. 1:**
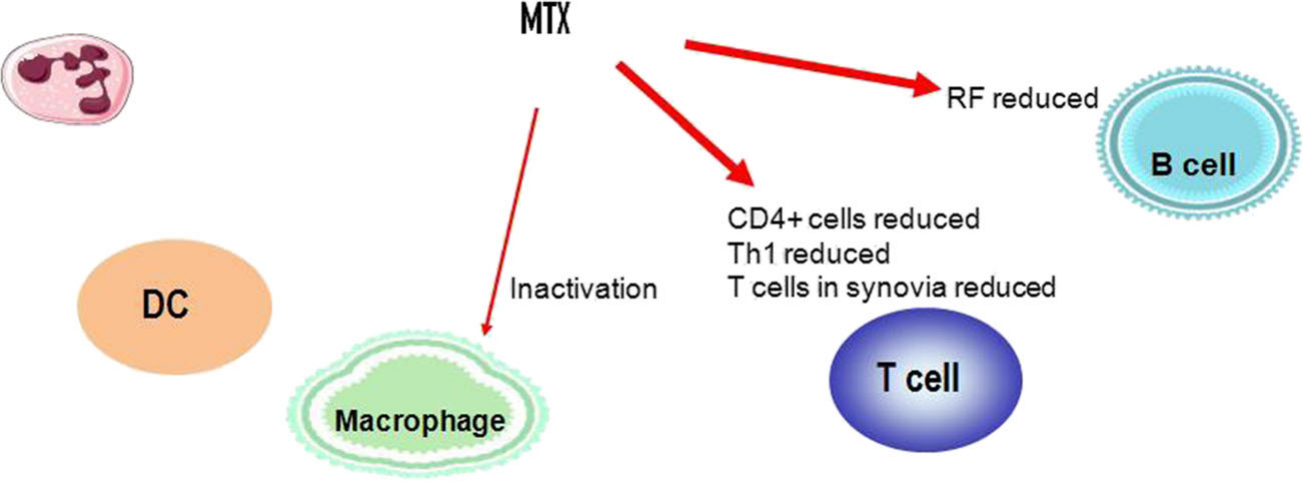
Immunological targets of methotrexate

In patients with RA, methotrexate inhibits the proliferation of cells and induces apoptosis (cell death) of activated cells. Both mechanisms mainly affect the lymphocytes and also, to a lesser degree, monocytes [17]. Methotrexate therefore reduces the number of activated T lymphocytes (Fig. 1). The expression of adhesion molecules such as ICAM-1 on T lymphocytes is reduced, as well as the production of the cytokines IL-6, TNF-α and GM-CSF by T lymphocytes [18]. The concentration of rheumatoid factor is also reduced in the process. In contrast to its effect on T lymphocytes, methotrexate does not substantially influence the cytokine production of monocytes [18].

Methotrexate probably reduces the interaction of T lymphocytes and synovial fibroblasts by reduced cytokine production, e.g. of IL-6, IL-15 and IL-17 [19]. On monocytes, the expression of Fc receptors I and II (CD64 and CD32) is decreased, so that the capacity of the monocytes to be activated by circulating immune complexes is reduced [20].

### Destruction of the joint

The production of RANK-L by synovial fibroblasts is decreased and that of osteoprotegerin is increased [21]. Both mechanisms reduce the differentiation of osteoclasts and, as a consequence, the amount of bone destruction. Methotrexate also reduces the concentration of MMP-1 and MMP-3 and thus could contribute to the preservation of the joint cartilage. In contrast, angioneogenesis does not appear to be influenced [22].

## Mode of action of the TNF inhibitors

As described above, TNF-α is one of the dominant cytokines in the pathogenesis of RA. TNF-α is predominantly produced by monocytes/macrophages, as well as by T cells, and activates, for example, macrophages and endothelial cells, after binding to the TNF receptor (CD120), which is expressed on multiple cells. As a consequence, there is an increase in the production of IL-1, IL-6 and other proinflammatory cytokines. Due to the important role of TNF-α in the pathophysiology of RA, TNF inhibitors are used in therapy. Infliximab is a chimaeric antibody targeted at TNF-α. Today, complete human antibodies against TNF-α are available in the form of adalimumab and golimumab, while certolizumab is a pegylated human anti-TNF-α antibody (which does not bind to Fc receptors); etanercept is a soluble TNF receptor. Everyday clinical experience shows that some RA patients respond to a second, but not the first, TNF blocker. Therefore, there may be subtle differences in the modes of action of the individual TNF inhibitors, which will not be discussed here in general, however.

TNF inhibitors influence the pathophysiology of RA on several levels.

### Adhesion/migration

At least in vitro, TNF inhibitors reduce the expression of VCAM on endothelial cells from the microvasculature of the intestine [23] and thereby reduce the ability of leucocytes to migrate from the blood into the inflamed tissue via adhesion to the vessel walls.

### Activation

**Fig. 2:**
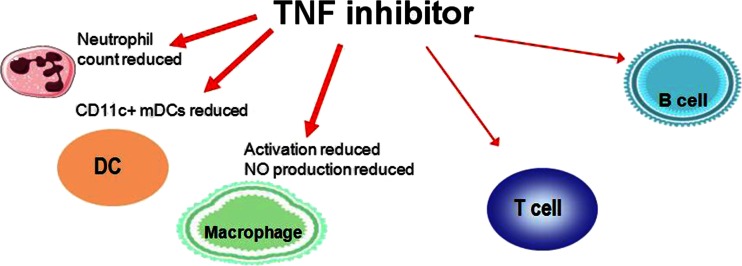
Immunological targets of TNF inhibitors

The degree of activation of the monocytes is markedly reduced [24, 25]. In the synovial fluid, the number of neutrophil granulocytes and myeloid dendritic cells (mDCs) is reduced during TNF inhibition (Fig. 2).

The influence on lymphocytes is less pronounced. In vitro studies have shown that infliximab is able to induce apoptosis (programmed cell death) at least in some T lymphocytes. In vivo, however, both infliximab and etanercept were able to trigger apoptosis only in monocytes, but not in lymphocytes [26]. The activity of CD4+CD25+-regulatory T cells which are involved in the protection from autoimmune disease is also unaffected [27]. The subpopulations of the B lymphocytes are generally not affected by TNF inhibitors. The number of memory B lymphocytes was increased only in the subset of RF-negative RA patients [28].

The change in cell populations affects the production of cytokines in the blood. Specifically, the concentration of proinflammatory cytokines produced by monocytes, such as IL-6, MIP-1 and CCL20 [29], is reduced during treatment with TNF inhibitors. The concentration of cytokines produced by T cells, however, does not change [29].

### Joint destruction

TNF inhibitors decelerate joint destruction even if the inflammation does not improve. TNF inhibitors reduce the production of MMPs (such as MMP-1, MMP-3, MMP-9) that contribute to the chondroclast activation and cartilage destruction [30]. Furthermore, the production of RANK-L and osteoprotegerin (involved with the osteoclast activation and bone destruction) is reduced [21, 31]. The production of VEGF, stimulating angiogenesis required for the blood supply of the pannus tissue, is also reduced [32, 33].

## Mode of action of tocilizumab

Tocilizumab binds both to the membrane-bound and soluble IL-6 receptor and can thus completely interrupt the effect of IL-6. IL-6 is a proinflammatory cytokine that binds to the IL-6 receptor and then to gp130, a signal transduction molecule. The membrane-bound IL-6 receptor is only expressed on few cell types. However, the soluble IL-6 receptor circulates in the blood and binds to IL-6. This complex can bind to gp130, which is expressed on the surface of most cells (*trans*-signalling). IL-6 therefore has a widespread effect on many cells and functions. In the inflammatory process, IL-6 increases the production of chemokines and expression of adhesion molecules on lymphocytes [34]. The production of chemokines (such as MCP-1, IL-8) is also increased in endothelial cells, rheumatoid arthritis fibroblast-like synoviocytes (RA-FLS) and mononuclear cells [35, 36], as is the synthesis of RANK-L by RA-FLS, and that of MMP-1, MMP-3 and MMP-13 in chondrocytes [37].

The costimulation of naïve T cells with IL-6 is key to their differentiation into proinflammatory Th17 cells, which play an important role in the pathophysiology of RA. IL-6 was originally identified as a differentiation factor of B cells and is involved in the development of plasma cells [38]. IL-6 also acts on plasmablasts and additionally induces the IL-21 production of CD4+ T cells [39], which in turn increases the proliferation of B cells.

### Activation

**Fig. 3:**
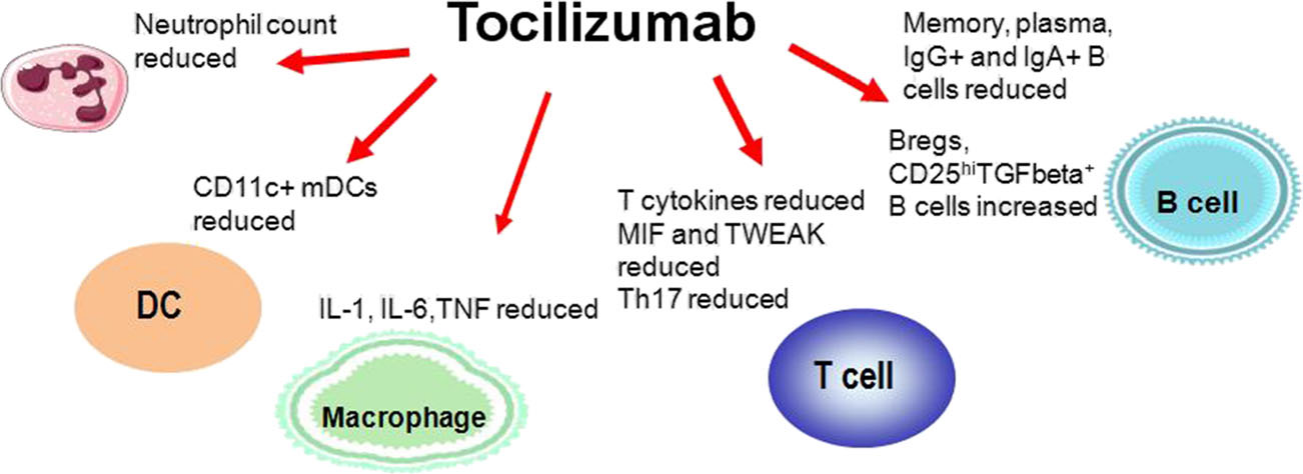
Immunological targets of tocilizumab

Tocilizumab has a particularly marked effect on the B cells. A reduction in the number of memory B cells [40, 41] has been seen in patients with RA and systemic lupus erythematosus (SLE), along with reductions in plasma cells [42] and increases of the number of regulatory B cells [43] that can interrupt immune reactions (Fig. 3). The number of B cells in the inflamed synovial membrane is reduced [44]. Tocilizumab can recover the T cell balance in RA patients as it reduces the number of Th17 cells and increases the ratio of regulatory T cells [45]. The concentration of the proinflammatory cytokines [29] produced by the T cells is also reduced. Tocilizumab not only acts on lymphocytes but also reduces the number of monocytes and myeloid dendritic cells in the peripheral blood of RA patients [46]. Additionally, the number of neutrophil granulocytes, which represent the majority of cells in the synovial fluid, is markedly reduced, at least in the peripheral blood.

### Joint destruction

Treatment with tocilizumab reduces VEGF and RANK-L production [47] by RA-FLS and thus angiogenesis and activation of osteoclasts. A reduction in RANK-L has been shown to increase the number of osteoblasts and reduce that of the osteoclasts in a monkey model [48].

A comparison of tocilizumab responders and nonresponders showed that the concentration of macrophage migration inhibitory factor (MIF), tumour necrosis factor-like weak inducer of apoptosis (TWEAK) and “C-X-C motif chemokine 10” (CXCL10) was reduced in responders [49]. MIF is produced by monocytes and T cells alike and is involved in the perpetuation of synovitis and destruction. During therapy with tocilizumab, proinflammatory cytokines such as IL-1 and IL-2 produced by T cells and TNF-α produced by monocytes are also reduced.

According to current insights, tocilizumab therefore has a very broad effect and regulates B and T lymphocytes, as well as the monocytes and dendritic cells.

## Implications for practice

- TNF inhibitors, whose mode of action (MoA) primarily targets monocytes and dendritic cells, and MTX, which inhibits lymphocytes, exert a synergistic therapeutic effect in RA.
- Tocilizumab has a broader effect than TNF inhibitors and mainly targets T and B lymphocytes, but also dendritic cells and monocytes.
- The combination of TNF inhibitors with methotrexate fills the gap of each substance with regard to the MoA, while tocilizumab alone has a very broad effect.
- The observation from studies, which show that tocilizumab monotherapy is as effective as tocilizumab in combination with methotrexate, is therefore not surprising.
